# Globally Critical Infrastructure: The Unique Risks and Challenges

**DOI:** 10.1111/risa.70147

**Published:** 2025-11-11

**Authors:** Zachary Kallenborn, Henry H. Willis

**Affiliations:** ^1^ Department of War Studies King's College London London UK; ^2^ Emerging Threats Group University of Oxford Oxford UK; ^3^ Center for Strategic and International Studies Washington USA; ^4^ Schar School of Policy and Government George Mason University Arlington Virginia USA; ^5^ RAND Center on AI, Security, and Technology Pittsburgh Pennsylvania USA; ^6^ RAND School of Public Policy Pittsburgh Pennsylvania USA

**Keywords:** critical infrastructure, global infrastructure, risk management, risk analysis

## Abstract

Critical infrastructure is typically identified at the national level. However, disruption to certain infrastructure systems, facilities, and assets can have negative consequences for global societies. Such globally critical infrastructure entails a distinct risk profile for both countries dependent on the infrastructure, and countries that have such infrastructure in their territory. The goal of the article is to provide an initial framing and definition of “globally critical infrastructure” as a concept worthy of attention and explore the unique risk analysis and management challenges to support future, more rigorous examinations. For dependent countries, globally critical infrastructure exists outside of their border (or possibly outside any country's border), under sometimes drastically different economic, political, governance, and threat environments. Risk management entails unique challenges, because countries dependent on that infrastructure may have no legal or regulatory authority to shape risk management practices at facilities in other countries. Consequently, risk management may extend beyond the domains of the typical homeland or internal affairs agencies to include capabilities and responsibilities of ministries of foreign affairs, trade and commerce, and defense. However, those challenges also imply new risk management demands and options, such as new avenues for international cooperation on infrastructure protection and resilience, international funding, and enhanced monitoring. Having a globally critical infrastructure system in its borders changes the risk dynamics for a nation state, creating potential leverage over dependent nations and new avenues to garner international support, but also creates new risks to national sovereignty. Recognizing these common dependencies can better enable the global community to engage stakeholders to develop and implement systemic risk management approaches worldwide.

## 1 Introduction

“Whatever highway may be constructed across the barrier dividing the two greatest maritime areas of the world must be for the world's benefit, a trust for mankind, to be removed from the chance of domination by any single power, nor become a point of invitation for hostilities or a prize for warlike ambition.” Former American President Grover Cleveland regarding ownership rights of a potential canal joining the Atlantic and Pacific oceans, December, 1885 (Quoted in Weld 1889)

Over the centuries, the international community has come together to establish norms and treaties to safeguard common interests and protect global commons. This has included the law of the sea, outer space law, the Antarctic treaty system, and developing numerous international fora to identify common opportunities, and litigate disputes. Yet, academic and policy discussions of critical infrastructure have largely focused on national policy. When global discussions occur, the focus tends to be on common themes experienced globally but manifesting primarily at the local or national levels. However, the strong and growing connections between global societies mean that common infrastructure dependencies are growing too. Consider the response to COVID‐19. In March 2020, two Pfizer facilities, one in Puurs, Belgium, the other in Kalamazoo, Michigan, were selected to manufacture COVID‐19 vaccines at industrial scale (Silver n.d.). Pfizer claims the two facilities produced 100 million doses per month collectively and have shipped vaccines to 166 countries (Silver n.d; Pfizer n.d.). The scale of production implies the global reach: just 1 year of production would generate enough vaccines for about 15% of the world's population.

However, no academic or policy analysis has focused on the challenges of identifying, assessing, and managing risks to such globally critical infrastructure. A Google scholar search for the term “globally critical infrastructure” on September 28, 2024 returned only 10 results, only one of which appears to use the term in the same sense as intended here. However, that study by Chris Demchak focused primarily on global cyber infrastructure (Demchak 2012). Google searches for the term “globally critical infrastructure” primarily return results of the form “globally, critical infrastructure” referring to global behaviors regarding critical infrastructure. Of those, two results are relevant. The first, a study on improving climate resilience to the Suez Canal offered general lessons to “other globally critical infrastructure assets” (Barnes et al. 2022). The second, the June 2020 meeting notes of NASA's International VLBI Service for Geodesy and Astrometry explicitly notes, “The concept of ‘critical infrastructure’ is only defined on a national level. There is no global equivalent” (Behrend 2020). Narrowed Google searches of “globally critical infrastructure” to UN.org, OECD.org, WEForum.org, the .gov and .eu domains, as well as prominent think tanks (RAND.org; RUSI.org; IISS.org; Chathamhouse.org) produced only one additional relevant document: a summary of a 2008 inspector general's report noting that the Department of Homeland Security international activities should include “better understanding globally critical infrastructure” (Committee on Homeland Security and Governmental Affairs 2008.)

Although several studies use the term “critical international infrastructure,” none appear to identify, define, and analyze the set as a common class, but rather use the term as a descriptor for specific types of infrastructure (see e.g., Scott‐Hayward 2024; Rapp et al. 2012; Ganz et al. 2024). The use of “international” also implies a different and much broader scope. Similarly, some studies use the term “global critical systems,” though again do not define or explore the systems as a comprehensive class (Mani et al. 2021; Kemp et al. 2022). Although it remains possible other studies use another term that has been explicitly defined and explored, we have been unable to locate them.

Nonetheless, specific forms of globally critical infrastructure do have existing literature, sometimes extensive, such as studies on the Suez (e.g., Lee and Wong 2021) and Panama canals, space infrastructure, cyber infrastructure (Clemente 2013), automobile manufacturing (Farooq et al. 2024), and trade chokepoints (Bailey and Wellesley 2017). Previously, the United States developed a list of foreign infrastructure it depends on through the Critical Foreign Dependencies Initiative, but items in this list may be unique dependencies for the United States and not necessarily global dependencies (Arce 2015). Further, the list is also no longer maintained and considered relevant, though some initial efforts to build a new version are ongoing (Interview With Former Department of Homeland Security Officials 2024).

Globally critical infrastructure is defined here as systems and assets, whether physical or virtual, so vital to societies around the world that the incapacity or destruction of such systems and assets would have a debilitating impact on numerous countries across multiple geographic regions (derived from The White House 2013). Disruption to such infrastructure could entail far greater and far broader consequences to many countries and to global society compared to national critical infrastructure disruptions.^1^ The nature of the systems entails unique risk analysis and risk management challenges, owing to a different range of threats and hazards, more complex stakeholder relationships, and differing national and global risk governance responsibilities and capabilities. Although infrastructure systems that could be considered globally critical have long been considered in isolation, defining and conceptualizing them as a common class has benefits for both fundamental understanding of policymaking and practical application for risk governance in both academia and government:

1. The common concept calls attention to the unique risk analysis and management challenges of this infrastructure class, which should be accounted for during risk analysis and management;
2. Recognizing the common geopolitical challenges of globally critical infrastructure may reveal novel opportunities for global collaboration and risk reduction, which, to the degree those opportunities do or do not emerge, deserves academic inquiry;
3. Focusing on common global infrastructure may call focused attention, research, and analysis into those systems, to include identifying systems whose attention is not proportional to their importance; and
4. Growing attention to global catastrophic risks like climate change and COVID‐19 as well as various long‐term oriented philosophies, suggests a need to focus attention on protecting and supporting humanity's common infrastructure.

To begin developing and exploring the globally critical infrastructure, this article will begin with a discussion of the emergence of globally critical infrastructure, including a framework for identifying such infrastructure. Then, the article will explore the unique risk analysis and management challenges, highlighting differing risk profiles, and the vastly more complex political and stakeholder environment. Next, the article discusses the potential policy implications implied in recognizing globally critical infrastructure as a unique set of systems, facilities, and assets meriting global attention. The conclusion offers next steps in developing and implementing the concept in real‐world policy.

## 2 The Emergence of Globally Critical Infrastructure

Globally critical infrastructure has become prominent because the world is far more interdependent and interconnected than it has ever been, and countries have encouraged and exploited global interdependence to advance policy and economic goals. In 2022, world trade volume was roughly 45 times higher than it was in 1950 (World Trade Organization n.d.). It is not just trade that is connecting the world.

A 2011 McKinsey report estimated that the Internet accounted for 10% of GDP growth from 1996 to 2011 among the most advanced economies, and the overall rate doubled from 2006 to 2011 to 21% (Manyika, and Roxburgh 2011). More recently, UN Trade and Development estimated that global e‐commerce jumped to $26.7 trillion in value during COVID‐19 (UN Trade and Development 2021). Global communities increasingly rely on the Internet for critical transactions, like paying rent or utilities, whereas global shifts to remote and hybrid work structures during COVID‐19 meant global commerce became even more dependent on Internet connectivity and enabling services like Zoom.

Similarly, space‐based communication and position, navigation, and timing services are critical for numerous aspects of global infrastructure. For example, the United Kingdom estimates that 17% of annual GDP is dependent on satellite services, and a 5‐days disruption alone could cause losses as high as 3.2 billion pounds (House of Commons Science and Technology Committee 2022). Other, traditional infrastructure sectors depend on space too. The National Institute of Standards and Technology has noted how time synchronization is critical for financial markets to manage incoming transactions, power grids to monitor real‐time loads and locate faults, and mobile communication networks require synchronized timing to ensure operations are synchronized (Lombardi 2021).

The global community has also increasingly recognized the world faces an array of potential globally catastrophic or existential risks from climate change to large magnitude volcanic eruptions and near‐Earth objects (Bostrom and Cirkovic 2008; Willis et al. 2024). Infrastructure to reduce these risks would necessarily be globally critical, because the risks they address are globally impactful. For example, the Svalbard Global Seed Vault in Norway is intended to provide a “global backstop” with seeds from tens of thousands of food crops from across the world (Ministry of Agriculture and Food 2007). Although disruptions to global seed stores may not be impactful in the near‐term, it could be globally catastrophic if disruptions correspond with a larger catastrophe where the seed stores prove critical, but unavailable. Likewise, active research and development activities seek to develop future infrastructure to, for example, cool super‐volcanoes to prevent catastrophic eruptions, and release particles into the atmosphere to reduce global greenhouse effects (Cox 2017). Once solar geo‐engineering to reduce global warming has begun, disruption could lead to rapid warming, creating greater global harms than climate change on its own, and also coming with potential opportunity costs of lost time and resources on alternative approaches to reduce climate change risks (Tang and Kemp 2021).

Potential examples of globally critical infrastructure span a broad and diverse range of systems, services, and assets. As a first step in advancing efforts to assess and govern risks of this type we offer a framework for identification grounded in definitions of three key terms: *infrastructure*, *critical*, and *global* and criteria for which each might lead an infrastructure to be considered globally critical. Anchoring governance of globally critical infrastructure on these definitions allows focused identification of cases that warrant special attention without distracting efforts away from the equally important domain of nationally critical infrastructure.

### 2.1 Defining Infrastructure

The Oxford English Dictionary gives the etymology of infrastructure as 1875, combining the Latin roots *infra* meaning below, or part of, and *structure*, “relating to the action or process of construction and to the condition or quality of being constructed.” (Oxford English Dictionary 2025) The term most commonly refers to “the basic systems and services … that a country or organization uses in order to work effectively” (Cambridge Dictionary 2025; Oxford English Dictionary 2025). Governments around the world have adapted this definition to include a variety of systems and services upon which society depends. For example, as shown in Table 1, the United States and European Union define an overlapping set of infrastructure sectors that span physical, economic, and societal systems that include intertwined logistical, transactional, and governance layers (Cybersecurity and Infrastructure Security Agency n.d.; European Commission 2025; Weber et al. 2023).

**TABLE 1:** Critical infrastructure sectors defined by the United States, the European Union, and a global review of 194 countries.

United States critical infrastructure sectors	European Union critical infrastructure sectors	Sectors identified through global review of critical infrastructure governance
Chemical Commercial facilities Communications Critical manufacturing Dams Defense industrial base Emergency services Energy Financial services Food and agriculture Government services and facilities Healthcare and public health Information technology Nuclear reactors, materials, and waste Transportation systems Water and wastewater	Energy Transport Banking Financial market infrastructure Health Drinking water Wastewater Digital infrastructure Public administration Space Production, processing, and distribution of food	Energy Information and communication technologies Health Transport Food Water Public services Economy and finance Research and education National security

National priorities for critical infrastructure management vary widely. A global review of 194 countries identified 100 published assessments of critical infrastructure. Focus on the problem varies widely across countries; 96% of countries in Europe and North America have published critical infrastructure lists compared to 42%, 49%, 29%, and 28% for Latin American and the Caribbean, Asia, Oceana, and Africa, respectively (Weber et al. 2023).

Similarities across the references included in Table 1 demonstrate some common understanding across nations. There is consensus on some sectors; such as energy, information and communications technology, transport, finance, government and administration, and health. Differences reveal variations in governance and oversight as well as underlying priorities. Sectors related to research and education, food, and water are included less consistently. In addition, sectors are organized differently in different regions. For example, the US sectors for communications and information technology are largely captured by the European Union digital infrastructure sector. In addition, the United States includes a nuclear sector, whereas the European Union includes a space sector. Accordingly, these lists have changed over time as priorities and shifts in governance structure resulted in inclusion, exclusion, or reorganization and renaming of sectors.

Although not typically included as critical infrastructure, some risk governance frameworks also recognize the importance of *green infrastructure* which provides:

“a strategically planned network of natural and semi‐natural areas with other environmental features designed and managed to deliver a wide range of ecosystem services… …[incorporating] green spaces (or blue if aquatic ecosystems are concerned) and other physical features in terrestrial (including coastal) and marine areas” (European Union 2013)

Inclusion of green infrastructure expands the systems to include resources that support global biomes and terrestrial, oceanic, and atmospheric systems. To the degree global focus on climate change continues to grow, emphasis on green infrastructure can be expected too, some of which may be globally critical like geo‐engineering systems.

When identifying globally critical infrastructure, it is important to consider elements that could exist across the broadest set of sectors that nations deem important.

### 2.2 Defining Criticality

Criticality stems from the benefits society obtains (or seeks to obtain) from infrastructure and the risk of loss when that infrastructure is disrupted, degraded, or destroyed. Empirical studies of risk perception reveal a range of outcomes that people care about related to health and safety (Fischhoff and Morgan 2013; Florig, et al. 2001), the environment and natural ecosystems (Willis et al. 2004), and homeland and national security (Lundberg 2013; Willis et al. 2018). Governments have focused these outcomes to encompass effects on national security, economic security, public health or safety, or any combination of those matters (The White House 2013). Because weighing such values is necessarily a subjective, often political process, actors will vary in the relative weight placed on different outcomes as well as the overall approach to identifying and managing risks. Social, political, and environmental changes may also change the relative weight countries and people place on those outcomes over time. In practice, some degree of at least limited consensus is required on what constitutes critically to support collective decision‐making.

In considering assessment of global catastrophic risks that could threaten human civilization or lead to human extinction, five dimensions of consequences from disruptions to globally critical infrastructure can be identified based on Willis et al. (2024):

- Mortality and morbidity: deaths and injuries resulting directly from infrastructure disruptions
- Economic instability: economic losses of varying degrees
- Governance instability: governance failures of varying degrees, to include threats to national security
- Ecosystem instability: degradation or destruction of the functions and services that support biomes and global terrestrial, oceanic, and atmospheric systems
- Reduced human capabilities: degradation of significant aspects of well‐being such as a general sense of security, justice and freedoms, and basic human needs and dignities (Nussbaum 2011).

On each of these dimensions, defining criticality requires considering criteria that would specify infrastructure as globally critical such as

- Intensity: the impacts in terms of all five dimensions of outcome, which could be reflected in both measures of magnitude and quality
- Geographic extent: how the effects span across national jurisdictional boundaries to extend across a region or subregion, a continent, a hemisphere, or globally
- Duration: do the impacts manifest over a period of months or less, years, decades, or centuries or more; including whether effects are expected to be permanent and irreversible.

Measurement of criticality will necessarily rely on mixed methods and interdisciplinary approaches. Some types of consequences are amenable to quantitative estimation; such as estimation of deaths and injuries, amounts of species or habitat affected, or economic losses. In cases where globally critical infrastructure is generated by interconnected systems, methods of graph theory and estimates of centrality can be leveraged (Dunn and Wilkinson 2012). Other types of consequences, such as instability of governance or reductions in human capabilities, may require reliance on qualitative analysis, indices, or information about public perceptions.

Thresholds for criticality will involve interactions among the factors of intensity, geographic extent, and duration. For example, a complete disruption of the global positioning system may not be significant if it only lasts fractions of a second, yet a permanent 25% reduction in the shipping capacity through the Suez or Panama canals could have immense economic impacts. Given tradeoffs across these factors and across consequences, the process of setting thresholds will require policymakers to capture and reflect value‐based priorities.

### 2.3 Defining Globally

In general, infrastructure that is globally critical will tend to be systems and assets around the world that numerous national‐level infrastructure systems and assets depend on for their own function, as well as systems and assets to address uniquely global challenges such as global governance institutions, security of global commons, and planetary defense and protection. Infrastructure may be globally critical because of three potential properties of an identified infrastructure.

First, the benefits of infrastructure could be the result of its *existence and operation as a global system*. This value exists beyond any value that might be generated by national or regional elements of the global system. For example, each nation has established financial networks to enable financial transactions, whereas the existence of the Society for Worldwide Interbank Financial Telecommunications (SWIFT) provides a unique service to provide messaging and transactions across national networks, as well as developing and propagating related standards (Scott and Zachariadis 2014).

Second, infrastructure may deliver a globally singular or distinctive service that is not easily replaceable. Examples include the Svalbard Global Seed Vault (i.e., food), the ITER magnetic fusion device in France (i.e., research), or the International Atomic Energy Agency (i.e., energy and public administration). Expanding the construct to include green infrastructure motivates inclusion of unique ecosystems and features that influence global ecosystems, such as the Amazonian forests, polar ice shelves, and infrastructure enabling activities like stratospheric aerosol injection (Reynolds 2019).

Finally, a specific infrastructure asset may serve a special role in a global system because of the connectivity it enables or the magnitude of the contributions it provides. For example, the Suez and Panama canals represent global chokepoints for global container shipping and Taiwan Semiconductor Manufacturing is responsible for 92% of global manufacturer of the most advanced semiconductors (Varas et al. 2021). Although disruption of chokepoint infrastructure like the Suez Canal may not completely end shipping, because ships could transit around, the increased time and costs could still create global disruption and impacts, especially for products dependent on parts or components to produce other goods.

These three properties illustrate that the network typology and, consequently, vulnerability and management, of globally critical infrastructure systems can vary greatly. Certain infrastructure like the canals is highly localized and bound to a geographic area. Meanwhile, certain global services could, in theory, be located anywhere, but may be difficult to reconstitute elsewhere for various political and economic reasons. Global systems like the submarine cable network are highly distributed with numerous potential points where disruption can occur.

“Globally” critical infrastructure can be understood as a subtype of foreign dependent infrastructure. As summarized in Table 2, national infrastructure is well‐recognized as having different geographic scopes from local water treatment plants to national‐level institutions like stock exchanges or defense industrial bases. Similarly, foreign infrastructure upon which a country depends can vary in geographic scope too from bilateral infrastructure where only one other country depends on it, to regional where multiple states in the same geographic region depend on it, to the focus of this study, truly global infrastructure. Note that the importance of foreign infrastructure will typically be simultaneously important through many types of dependency relationships. As a result, global infrastructure may also carry importance at bilateral, regional, and national levels. For example, Taiwanese semiconductor manufacturing is global infrastructure that is also important foreign infrastructure at the regional level (the European Union), and the bilateral level (e.g., specific exchanges between the United States and Taiwan), as well as serving as nation‐wide infrastructure for Taiwan.

**TABLE 2:** Level of infrastructure.

Infrastructure level	Sublevel	Example
**National**	Local	Municipal water treatment facility
	Regional	The Hoover dam
	Nation‐wide	National stock exchange
**Foreign**	Bilateral	United States–Canada integrated power grid
	Regional	Nord Stream pipeline
	Global	Taiwanese semiconductor manufacturing companies

Of course, nations may differ in their degree of dependence on any particular globally critical infrastructure system, so disruption might have disproportional effects. Some countries might be affected extremely by disruptions, whereas others might not. For example, a highly digitized society is likely to be far more affected by disruptions to semiconductor production compared to countries with very limited access to or use of computers.

### 2.4 Identifying and Describing Globally Critical Infrastructure

These definitions and dimensions provide a framework that could facilitate identification and characterization of globally critical infrastructure. Table 3 provides a list of examples of infrastructure and the reasons each infrastructure may be considered globally critical. These characterizations can in turn provide a basis from which to consider analyzing and managing globally critical infrastructure.

**TABLE 3:** Examples of globally critical infrastructure.

		Relevant dimensions of criticality				
Global nature of infrastructure	Examples of infrastructure sector/asset	Morbidity and mortality	Economic stability	Governance stability	Ecological stability	Enablement of human capabilities
Global system	Space launch (transport)		X	X	X	X
	SWIFT (Finance)		X			
	GPS (space)	X	X	X		
	Undersea cable network (telecommunications)		X	X		
Singular global service	Svalbard Global Seed Vault (Food)	X			X	
	ITER (research/energy)		X			X
	International Atomic Energy Agency (energy/government)	X	X	X		
	Amazonian forests (green infrastructure)	X	X		X	X
	World Trade Organization (Government)		X	X		
	Gavi Vaccine Alliance (Healthcare)	X				X
Global chokepoints	Suez Canal (transport)		X	X		
	Taiwan semiconductor manufacturing companies (manufacturing)		X	X		
	Ultra‐high purity quartz mines (critical minerals)		X	X		

As can be seen, numerous potential examples of globally critical infrastructure exist, and many contribute to several dimensions of critical outcomes, albeit to differing degrees. Notably, almost every identified infrastructure system contributes to economic outcomes, which reflects the observation that the drivers criticality for much of what could be considered globally critical infrastructure is the pervasiveness of global trade and economic interconnectivity. Further, the list also illustrates the broad range of globally critical infrastructure, as the list covers most forms of traditional infrastructure.

## 3 Analyzing Risk to Globally Critical Infrastructure

Kaplan and Gerrick (1981) describe risk analysis as answering three questions: “(i) what can happen? (i.e., what can go wrong?); (ii) how likely is it to happen?; and (iii) If it does happen, what are the consequences?”. On all three questions, globally critical infrastructure has major differences from typical national critical infrastructure.

A defining feature of globally critical infrastructure is that the effects of disruptions extend far beyond national borders. That means priority threats and hazards for globally critical infrastructure will often differ from (though may still overlap with) those that affect national infrastructure, along with associated likelihood assessments. For example, although an infrastructure‐hosting and infrastructure‐dependent country may face terrorist threats, the ideology, technical capacity, and preferred tactics may differ drastically. Similarly, infrastructure‐hosting states may differ in their regime type, economic system, degree of governmental and private sector corruption, human rights record, regulatory and policy environment, national control over infrastructure, geopolitical landscape, threat actors, and natural hazards all of which may affect the successful operation of the infrastructure system. For example, the five biggest importers of Saudi Arabian oil are China, India, Japan, South Korea, and the United States (Observatory of Economic Complexity n.d.). None of the five are monarchies located in the Middle East facing direct threats from Iran and regional proxies. In the 2019 Abqaiq–Khurais attacks, Iranian drone strikes on Saudi Aramco oil refining facilities resulted in a 50% cut in Saudi oil production, amounting to 5% of global oil production (BBC 2019). Although China, India, Japan, South Korea, and the United States may not worry about Iranian drone attacks on their national infrastructure, the threat still had and could have a significant impact on infrastructure operations.

On the one hand, the global dependence of infrastructure means an actor could harm numerous adversaries by disrupting this infrastructure to reduce the magnitude or quality of services that it provides. For example, if Russia wanted to harm American trade, disrupting the Panama Canal would certainly be extremely impactful. On the other hand, common, global dependencies on the infrastructure may generate self‐deterrence effects. Russian ships also travel through the Panama Canal, so disruption would affect trade there too (France24 2022). However, conversely, globally critical infrastructure might attract the attention of actors who may be delighted to generate global harm, either as part of an asymmetric coercion strategy or to achieve larger ideological gains. Various extremist groups, for example, increasingly hold accelerationist ideologies focused on generating larger social collapse. Those organizations have and will likely continue to focus on targeting critical infrastructure to radicalize and recruit members, advance their narratives, and generally cause damage, because infrastructure is a necessary component of maintaining modern society (Clarke 2023). All this means states providing globally critical infrastructure may face unique threats to their sovereignty from more powerful states and a range of other hostile actors.

The different risk sets also imply different organizations are needed to conduct risk assessment, particularly with a much greater emphasis on agencies that enable shared awareness among nations. A country dependent on a globally critical infrastructure system may have limited insight into the risk management activities of other countries, except to the degree the country elects to share them. Countries providing the infrastructure may be quite hesitant to share risk‐related data, because it may be security sensitive, economically sensitive, or fear sharing the information may prompt undesirable actions. This is especially likely to be the case regarding internal security threats that may upend or disrupt governmental functioning.

By definition, many countries depend on globally critical infrastructure, and that infrastructure will rarely (and perhaps never) exist within their national borders. The external locus of control implies that states need to assess risks to globally critical infrastructure upon which they are dependent seperate from national critical infrastructure. Researchers have concluded that the most important factor in realizing the economic benefit of the Suez Canal is the stability of the Egyptian regime (Chorev 2023). So, Suez risks might include factors like Egyptian monetary and fiscal policy; human rights records; and democratization (Zaki 2017; Mansour 2025). Consequently, risk assessment will also require different types of regional and functional subject matter expertise to appropriately assess and account for the larger range of risks. That will require the risk assessing state to either develop the indigenous knowledge and capability to critically assess such risks, or build relationships with other states, such as the infrastructure provider, who can share that knowledge. However, as the Suez illustrates dependent states may have mismatched risk perceptions: For European countries and others, the Suez is external, global infrastructure, but for Egypt it is also critical national infrastructure.

Even small disruptions to globally critical infrastructure may be globally consequential and major disruptions could amount to a global catastrophe, especially when globally critical infrastructure systems are geographically concentrated (Mani et al. 2021). In March 2021, the Ever Given container ship became trapped in the Suez Canal for 6 days (BBC 2021). Lloyd's list estimates the ship held up $9.6 billion per day in global trade, while the Wall Street Journal notes it simultaneously worsened shortfalls in critical goods like semiconductors (Harper 2021; Stahl 2021). The event generated global headlines even months later (Yee and Glanz 2021). Similarly, disruption to mass vaccine production facilities just before or in the midst of a new global pandemic could result in millions more people dying who might not otherwise would have, because vaccines are not available or achieving herd immunity is postponed. Although not every disruption may be so impactful and not every infrastructure system is so sensitive to disruption, the growing interconnectivity of infrastructure means disruptions may have significant consequences in unexpected ways. Influential risk researcher Yacov Haimes noted that modern societies are complex large‐scale system of systems: “Each system is composed of numerous interconnected and interdependent cyber, physical, social, and organizational infrastructures (subsystems), whose relationships are dynamic (i.e. ever changing with time), non‐linear (defeating a simplistic modeling schema), probabilistic (fraught with uncertainty), and spatially distributed (agents and infrastructure with possible overlapping characteristics are spread all over the continent(s)).” (Haimes 2008) This complexity coupled with worldwide dependence on globally critical infrastructure means that disruptions can be expected to have major downstream consequences on other forms of critical infrastructure. Even if most countries have adequate preparedness and resilience to handle the effects of a disruption, some may not. Simultaneous disruptions to multiple globally critical infrastructure systems could be especially harmful, as disruptions combine in uncertain and complex ways.

## 4 Managing Risk

Haimes (1991) offers another set of three questions to capture risk management: “What can be done? What options are available and what are their associated trade‐offs in terms of all costs, benefits, and risks? And what are the impacts of current management decisions on future options?” Again, globally critical infrastructure will have unique answers to all three questions.

Traditional domestic policy, regulatory, and legal tools may have little value in encouraging or coercing protective behavior for globally critical infrastructure that operates in another jurisdiction. Consequently, traditional, domestic‐focused critical infrastructure bureaucracies may be limited, instead shifting responsibility to externally focused ministries of foreign affairs, trade and commerce coordination, and defense.

Similarly, the benefits from, responsibilities for, and capacity to steward globally critical infrastructure might not align well with national boundaries. By its nature, globally critical infrastructure affords benefits that extend beyond national borders. This could create responsibilities for nations that host globally critical infrastructure, while also creating interests and associated responsibilities for other countries. The resources available or priorities within the host country may not support or align with sustaining the globally desired infrastructure to meet the interests of other states. Aligning these interests, responsibilities, and capacities adds additional complexity to managing globally critical infrastructure and several considerations related to infrastructure as a tool of global power, implications of infrastructure and conflict, governance structures for risk management, and coordination of risk management.

### 4.1 Infrastructure as Global Power

Hosting globally critical infrastructure creates opportunities for states to use infrastructure as a tool of geopolitical power (This point is made well in Farrell and Newman 2019). Global dependency on infrastructure gives the owner/operator of the infrastructure system some power over dependent states. An owner/operator may revoke access to one or more states as a coercive or punitive measure to support the owner/operator's goals. For example, in April 2023 Kazakhstan seized Russian property at the Baikonur Cosmodrome, the world's largest spaceport, and barred Russian leadership from leaving the country, because Kazakhstan demanded $26 million in unpaid debts (Eckel 2023). The seizure opened questions about Kazakhstan's continued support to create the new Zenit‐M space launch facility, necessary for Russia's launch of new Soyuz‐5 rockets (Eckel 2023). Similarly, in February 2022 Turkey closed the Bosphorus and Dardanelles straits to warships in opposition to Russia's war with Ukraine, though it is unclear whether all warships or just Russian and Ukrainian warships were banned (Mongilio 2022; Pedrozo 2022). Article 19 of the 1936 Montreux Convention which governs transit through the straits allows Turkey to prohibit access to warships in conflict, and all warships if Turkey is party to the conflict (Pedrozo 2022).

Fears of infrastructure being used as a form of power may also motivate states to decouple from globally critical infrastructure. This is perhaps most notable in the case of North Korea, whose governing ideology is based on *Juche* philosophies of self‐reliance in political, economic, and military matters (Kim 2017). Similarly, how and whether Europe should divorce Russian natural gas dependence continues to be a fraught political issue (Gross and Stelzenmüller 2024).

### 4.2 Infrastructure and Conflict and Cooperation

Risk management for globally critical infrastructure can become major matters of global conflict. On October 29, 1956, Great Britain, France, and Israel launched a combined attack on Egypt following Egypt's nationalization of the Suez Cana (Wright et al. 1980). In 1885, the United States supported a rebellion in Panama pushing for independence from Colombia in large part because the United States desired control over the yet‐to‐be‐built Panama Canal (Wicks 1980). Then during Operation Just Cause in 1989, the United States invaded Panama, in part, to “protect the integrity of the Panama Canal Treaty” after General Manuel Noriega declared a military dictatorship (Bush 1989). Although the United States did not respond militarily following the September 2019 attacks on Saudi oil processing facilities at Abqaiq, former President Trump stated the United States was “locked and loaded” and issued a new round of economic sanctions on Iran (Rampton and Mohammed 2019). Other examples exist too.

The presence of globally critical infrastructure in a state party to a conflict may also change the geopolitical dynamics of that conflict. Taiwanese semiconductor facilities have been described as a potential “silicon shield,” because they would provide a strong incentive for the United States to support intervention in a conflict with China on Taiwan's behalf (Lee et al. 2021). Similarly, US security guarantees to Saudi Arabia have long been recognized to be a product of Saudi Arabia's criticality to American and global oil production (Spalding 2023).

But globally critical infrastructure is not just a cause of conflict. The infrastructure may also present diplomatic opportunities to improve relations. Russia–United States collaboration on the International Space Station has been a hallmark of science diplomacy for decades. Increasing the resilience of commonly shared globally critical infrastructure could be an opportunity to broaden those benefits. When risks manifest, the collaborative response can catalyze larger diplomatic efforts for peace and security (Kelman 2018). Of course, geopolitical sensitivities may preclude cooperation on threats or infrastructure with strong security relevance; however, opportunities might exist for security‐neutral resilience, such as improving canal resilience to natural hazards. Certain common threats may also justify cooperation on security‐sensitive issues, such as US‐Russian counter‐terrorism intelligence sharing (McDermott 2004).

### 4.3 Governance Structures for Risk Management

The global nature of globally critical infrastructure risk means a much broader range of stakeholder groups will be involved in analyzing, mitigating, and governing risk. By definition, a broad range of states, international organizations, multinational corporations, nongovernmental organizations, and others will have a stake in the functioning of globally critical infrastructure. On a day‐to‐day basis, these actors may not concern themselves with the infrastructure's operations, as they may have other priorities; however, disruptions to effective operations may generate global concern and attention. Some globally critical infrastructure may also be inherently transnational, such as undersea cables which may originate and terminate in two separate countries, subjecting the cable to different terminology, regulations, permitting, and other factors (Bowden et al. 2024). The broad stakeholder interest may also lead to novel risk governance approaches, such as the 1956 proposal for an international “Suez Canal Users’ Association” comprised of 15 countries with a common interest in the canal (UK Parliament 1956).

Managing risk to globally critical infrastructure will also necessarily imply focus on a different set of infrastructure systems, facilities, and assets. After WikiLeaks released a classified list of American critical foreign dependencies, economist Daniel Arce analyzed the list and concluded that “what the United States identifies as critical international infrastructure differs significantly from what is defined as the national critical infrastructure” (Arce 2015) Managing risk to globally critical infrastructure can be expected to require a significant expansion of managed systems, facilities, and assets, necessarily requiring new resources.

Managing risks to globally critical infrastructure is likely to involve different organizational equities too. Infrastructure protection is typically the remit of global ministries of internal affairs/security. In the United States, for example, the Department of Homeland Security's Cybersecurity and Infrastructure Agency leads interagency efforts to reduce critical infrastructure risk, whereas Department of Defense plays a supporting role in certain areas around homeland defense and the defense industrial base. This is appropriate, because a country's critical infrastructure typically falls under the jurisdiction of that country's legal, regulatory, and policy architecture. However, globally critical infrastructure may be owned and managed by other countries. As such, ministries of foreign affairs become essential, because any influence (or not) that a state may have over another will necessarily exist in the context of the overall relationship between the two states. If one state is concerned that another is leaving open vulnerabilities to global critical infrastructure upon which the first depends, that issue will need to be addressed through formal diplomatic channels. Similarly, because globally critical infrastructure is “globally” critical, other states are likely to take an interest in risk management policies and procedures. That means if a state has concerns, they may also need to navigate a complex geopolitical environment. International affairs offices within ministries of interior affairs might be sufficient for some bilateral or even multilateral discussions, but formally trained diplomats and appointed ambassadors will be necessary at times to navigate the political complexities.

In practice, the broad range of organizational equities means risk mitigation is likely to require significant interagency and international coordination. Homeland and internal affairs ministries responsible for national‐level critical infrastructure will be necessary to anticipate the impacts of a disruption on national‐level infrastructure; however, collaboration with academia, nongovernmental organizations, and international partners may be necessary to fully understand and anticipate the effects, because second‐ and third‐order effects are likely. For example, it should be easy to determine the value of trade in a given country that flows through the Suez Canal; however, trade in critical parts or materials needed for a downstream manufacturer may be disrupted too. Close communication and collaboration between private and governmental critical infrastructure owners and operators at the inter and intrastate level are likely to be necessary. All those perspectives need to be linked with defense, foreign affairs, and trade perspectives to develop aligned and comprehensive programs, policies, and strategies.

### 4.4 Coordination of Risk Management

Coordinating risk reduction will also be a challenge because states that own and are responsible for globally critical infrastructure may have different risk perceptions compared to dependent countries. External states that depend on the infrastructure may be narrowly interested in the protection of that infrastructure, whereas the states where infrastructure is located must juggle a range of competing domestic and international priorities. The states where infrastructure is located will necessarily have limited budgets for infrastructure protection activities and may choose to prioritize other, national‐level infrastructure. Differing priorities could and have emerged as sources of conflict between infrastructure dependent and infrastructure‐providing nations.

Infrastructure‐dependent states may also face internal tensions between risks management, and the larger foreign policy goals of the dependent states. During the Suez Crisis of 1956, the Eisenhower administration in the United States feared the British‐French‐Israeli invasion of Egypt would prompt a Soviet intervention, ultimately leading to greater Soviet influence in the Middle East (Department of State n.d.). The United States supported a UN resolution denouncing the invasion, and pressured Britain and France to accept a ceasefire (Department of State n.d.). The United States paid a price for the approach in temporarily damaged relations with Britain and France (Department of State n.d.). These competing priorities may also create decision‐making challenges if critical leaders weigh the risks differently.

Of course, risk management and larger foreign goals are not inherently at odds. Positive relations between states on risk management could bleed into larger collaborative relationships that strengthen interstate ties. For example, the United States Department of State and Homeland Security might collaborate to support Taiwan in building a national exercise program and improving its emergency response system, which could help reduce risk to semiconductor protection while also supporting Taiwanese emergency management more generally and strengthening bilateral ties (Bosner and Chang 2020). Likewise, the Odyssean Institute proposed the use of preferential trade agreements to enhance global trade resilience (Odyssean Institute n.d.). And even when there is conflict, it may not rise to a major crisis.

Another challenge is coordination across potentially numerous public–private entities. Aggregated infrastructure systems like the Internet depend on the successful operation of a multitude of physical and digital infrastructure and enabling services owned and managed by a multitude of governmental and private sector entities. These include data centers, subsea communication cables, Internet exchange points, Internet service providers, and management of the domain name system. More broadly, non‐state actors typically own and manage globally critical infrastructure, and their activities may be covered under multiple legal jurisdictions. Thus, risk management takes place in a highly complex stakeholder environment, and stakeholders may hold different values, and risk perceptions (Luhmann 1990). In turn, that may lead to underinvestment and free‐riding, as actors prioritize values other than security and risk.

### 4.5 Capacity Differences

Infrastructure‐hosting and infrastructure‐dependent countries can also be expected to differ, sometimes significantly in their capacity to manage risks. Numerous countries all around the world depend on globally critical infrastructure, to include countries that may lack significant economic, technological, military, and governance capacity. If a less powerful state is concerned about threats to global infrastructure they depend on, they may have few tools available to encourage or influence risk management practices. Conversely, infrastructure‐hosting countries may have limited capacity to address the risks they face. Although the legacy of post September 11th counter‐terrorism means the United States faces limited direct threats from sophisticated, hierarchical terrorist organizations, Middle Eastern states like Egypt and Saudi Arabia face major direct regional threats from Iran‐sponsored terrorist organizations like Hamas, Hezbollah, and the Houthi rebels. Those terrorist groups pose significant threats comparable to state‐level militaries, and consequently, Egypt and Saudi Arabia have often relied on help from the United States to repel the threats. Similarly, countries may differ in the broad legal, regulatory, and governance regimes to support risk reduction, that may either restrict or enable approaches. For example, countries with greater nationalization and state control of the private sector may be able to insist on infrastructure providers adopting various measures that countries with greater public–private separation could not.

## 5 Conclusions/Next Steps

In summary, globally critical infrastructure entails unique risk assessment and management challenges. The biggest of which is that infrastructure providers reside outside the control of states dependent upon them. This means the sets of hazards facing this infrastructure, the tools for managing them, and the stakeholders involved will all differ from traditional risk management. The consequences of disruptions will often be quite high, because harms will spread to multiple sectors in multiple nations, creating high potential for cascading consequences. Unfortunately, global governments and civil society are not looking at this class of infrastructure in a comprehensive way. So, what should academics and policymakers do now?

To further develop the concept, a critical next step is to develop a formalized approach to more precisely, and systematically identify globally critical infrastructure across not only traditional infrastructure sectors, but also infrastructure that may transcend typical infrastructure boundaries and is necessary for, but not typically considered, as critical infrastructure (e.g., international standards bodies). Developing a common list of globally critical infrastructure is likely to be difficult, but not insurmountable. First, a methodological issue exists of determining the threshold for “global” criticality as opposed to simply national or regional. Waterways like the Jordan River might be regionally important to states relying on it, but be minimally important globally. However, a globally critical infrastructure system will almost certainly be national and regional in importance. Second, states may be unwilling to share information about the infrastructure they depend upon for fear international rivals may misuse the information.

In practice, identification of globally critical infrastructure will almost certainly be a political process rather than an academically robust methodology. One potential solution is for global criticality to be self‐nominated with the global community accepting or rejecting that nomination. Assume for the sake of argument that the United States considers the Panama Canal a critical foreign dependency, and that determination is classified information. If the government of Panama nominated the Panama Canal as globally critical and provided a case why, the United States could choose to support (or not) based on the merits of the case and the larger international politics. The United States might choose to support Panama's bid ostensibly either to support bilateral ties, support allies, or simply because Panama made a good case that they are important, without acknowledging the classified determination that the United States is deeply dependent on the canal. American diplomats might also work quietly behind the scenes to encourage either Panama making its case, or others to support the bid. Although the specifics should depend on context, at face value, a self‐nomination approach seems to provide numerous means for states to create plausible deniability regarding their interest in a particular globally critical infrastructure system. States in which globally critical infrastructure exists would also have an incentive to seek designation, because it may unlock various international economic, political, and technological support to improve their country. In practice, sympathetic global governments could convene international tiger teams of infrastructure and foreign policy experts as part of multilateral fora like the G‐7 or five‐eyes to explore common approaches to identifying and governing globally critical infrastructure.

Nonetheless, academic insight may still have a role in attempting to identify this infrastructure in a systematic and rigorous way. In fact, academic‐led work could also be a simple solution to the information sharing problem in providing a common, open‐source baseline that states can react to. Academics thinking about and debating how criticality and global‐ness manifest could provide useful insight into policy‐development processes, as well as developing novel ideas around risk governance.

At the national level, states will differ in their degree of dependency on any particular globally critical infrastructure, national‐level efforts are needed to determine which infrastructure that particular country is dependent on and the country's general strategic approach to ensuring access and reducing risk. Nationally identified infrastructure should receive dedicated risk analysis to account for the broader scope of hazards that might exist with appropriate inclusion in national risk registers. This should include scenario analyses and sensitivity analyses to better characterize how risks may manifest, and their consequences for the national system. States should also create or designate an entity to lead risk analysis and management efforts around globally critical infrastructure. For example, the United States might designate the Department of State or Commerce as the lead Sector Risk Management Agency for globally critical infrastructure, responsible for leading interagency coordination and collaboration (Cybersecurity and Infrastructure Security Agency n.d.). This would ensure risk management activities are conducted consistent with and supporting larger foreign policy goals. Alternatively, because globally critical infrastructure necessarily crosscuts traditional infrastructure sectors, the American National Security Council could establish an interagency policy committee that includes representatives from the Departments of State, Defense, and Intelligence Community to develop an approach to governing and managing risk to globally critical infrastructure.

Countries should also collaborate at relevant international fora to identify and assess common dependencies among members, and collaborate to develop, implement, and improve risk management approaches. For example, the final assessment report of an EU‐NATO task force recommended: “Developing regular Parallel and Coordinated Assessments of the threats to critical infrastructure…” which could include threats to globally critical infrastructure of interest to the EU and NATO (NATO 2023). Similarly, the Organisation for Economic Co‐Operation and Development (OECD) might raise the topic in the High‐level Risk Forum to identify common infrastructure dependencies among member states and identify and develop common governance strategies. The OECD might also coordinate tailored risk assessments of specific globally critical infrastructure systems and identify opportunities for member engagement. International defense fora like NATO and five‐eyes intelligence sharing community may also allow for more candid discussion of classified assessments and categorizations of globally critical infrastructure. At the United Nations, the United Nations Security Council could order reports on global security threats to globally critical infrastructure, whereas the United Nations Disaster Risk Reduction agency could create a funding stream dedicated to reducing natural hazard risk to globally critical infrastructure. The G20 is also a potentially useful forum for raising the topic of globally critical infrastructure, owing to their broad participation from both states and civil society and on‐going work on global infrastructure generally.

Given that so much of global infrastructure is owned and operated by private sector entities, the private sector will need to play a leading role. There is a solid basis for doing so. Although the American government led early development of the Internet, Internet governance has become largely privatized (Sherman 2020). Governments continue to exercise influence through laws, building infrastructure, and setting norms and standards, but private sector entities typically finance and develop subsea cables, data centers, and cloud services all of which affect both the structure of the Internet and risks to it (Sherman 2020). Insights from experiences governing risk to Internet infrastructure and working across the public–private divide may be useful for reducing risk to other globally critical infrastructure.

Another specific group of entities who should play a role is international development agencies and banks. States use multilateral development banks to fund and support development in middle and low‐income countries, which has and could include infrastructure projects (Avellán et al. 2024). Globally critical infrastructure exists not only in developed states, but middle‐ and low‐income states too. Providing funding to those states to help support various risk and disaster management activities around globally critical infrastructure would be valuable not only for the middle‐ and low‐income states but also for the global community. International development agencies and banks should create dedicated funding streams for this purpose to encourage and solicit support from wealthy nations.

Globally critical infrastructure should also be considered in relevant hazard‐specific analyses at all levels. It is especially critical to explore scenarios where the same hazard—or some combination of hazards—harms multiple globally critical systems, facilities, and assets, because that could create compounding and cascading consequences. One obvious hazard worth focus is climate change. Climate‐induced hazards like rising sea‐levels, more frequent and higher intensity natural hazards, and global instability could potentially impact a broad range of globally critical infrastructure, compounding larger threats to international economic and social systems.

Finally, a new focus on globally critical infrastructure may open the aperture of critical risk management to global systems not typically considered infrastructure, such as green infrastructure as discussed above. Another possibility is the global system of scientific discovery and innovation. Considering scientific discovery and innovation as a critical function of critical infrastructure has some basis: In the American Cybersecurity and Infrastructure Security Agency's National Risk Management Center designated “research and development” a national critical function, whereas the German Federal Agency for Cartography and Geodesy has pushed to designate the Geodetic Observatory Wettzell as critical infrastructure (Behrend 2020). Scientific discovery certainly has implications for all relevant dimensions of criticality. Without the germ theory of disease and the discovery of bacteria and viruses, humanity would have been ill‐equipped to respond to COVID‐19 or any other pandemic; the economic benefits of the Internet and digitization could not exist without the development of computers. Although immediate harms may be minimal except for certain facilities or in certain circumstances (e.g., disruption to the ability of scientists to share information about a new pathogen right when a pandemic breaks out), long‐term consequences could be catastrophic. What happens if a supervolcano erupts before humanity can discover how to prevent it or respond?

Today, global societies are more interconnected than ever before. But greater interconnectivity means greater interdependency and vulnerability. Critical infrastructure risk analysis and management needs a paradigm shift to look beyond national borders to the infrastructure upon which all of us depend.

## Conflicts of Interest

The authors declare no conflicts of interest.

## Data Availability

Data sharing is not applicable to this article as no new data were created or analyzed in this study.

## Endnotes

1 We use “disruption” as a catch‐all term to include any event preventing infrastructure from generating desirable outcomes. In practice, we expect differing risk dynamics between the physical destruction of infrastructure, narrowly operational disruptions (e.g., a temporary power outage), loss of social trust in infrastructure systems, and other events; however, we reduce these all to “disruption” for the sake of linguistic simplicity.
